# Are HIV positive patients resistant to statin therapy?

**DOI:** 10.1186/1476-511X-6-27

**Published:** 2007-10-24

**Authors:** Kevin W Johns, Matthew T Bennett, Gregory P Bondy

**Affiliations:** 1 Department of Medicine, University of British Columbia, Vancouver, Canada; 2 Immunodeficiency Clinic-HIV Metabolic Clinic, St. Paul's Hospital, Vancouver, Canada

## Abstract

**Background:**

Patients with HIV are subject to development of HIV metabolic syndrome characterized by dyslipidemia, lipodystrophy and insulin resistance secondary to highly active antiretroviral therapy (HAART). Rosuvastatin is a highly potent HMG-CoA reductase inhibitor. Rosuvastatin is effective at lowering LDL and poses a low risk for drug-drug interaction as it does not share the same metabolic pathway as HAART drugs. This study sought to determine the efficacy of rosuvastatin on lipid parameters in HIV positive patients with HIV metabolic syndrome.

**Results:**

Mean TC decreased from 6.54 to 4.89 mmol/L (25.0% reduction, p < 0.001). Mean LDL-C decreased from 3.39 to 2.24 mmol/L (30.8% reduction, p < 0.001). Mean HDL rose from 1.04 to 1.06 mmol/L (2.0% increase, p = ns). Mean triglycerides decreased from 5.26 to 3.68 mmol/L (30.1% reduction, p < 0.001). Secondary analysis examining the effectiveness of rosuvastatin monotherapy (n = 70) vs. rosuvastatin plus fenofibrate (n = 43) showed an improvement of 21.3% in TG and a decrease of 4.1% in HDL-C in the monotherapy group. The rosuvastatin plus fenofibrate showed a greater drop in triglycerides (45.3%, p < 0.001) and an increase in HDL of 7.6% (p = 0.08).

**Conclusion:**

This study found that rosuvastatin is effective at improving potentially atherogenic lipid parameters in HIV-positive patients. The lipid changes we observed were of a smaller magnitude compared to non-HIV subjects. Our results are further supported by a small, pilot trial examining rosuvastatin effectiveness in HIV who reported similar median changes from baseline of -21.7% (TC), -22.4% (LDL-C), -30.1% (TG) with the exception of a 28.5% median increase in HDL. In light of the results revealed by this pilot study, clinicians may want to consider a possible resistance to statin therapy when treating patients with HIV metabolic syndrome.

## Background

Patients with HIV are subject to dyslipidemia and other complications secondary to their highly active antiretroviral therapy (HAART) that are often classified as HIV metabolic syndrome [1-3]. As a result, these patients are at an increased risk for cardiovascular events such as myocardial infarction [4-6]. Currently pravastatin, atorvastatin and fluvastatin are recommended for treatment of HAART-related dyslipidemia as they pose a low-risk of pharmacological interaction with ongoing HAART [7,8].

Rosuvastatin is a highly potent 3-hydroxy-3-methylglutaryl coenzyme A (HMG-CoA) inhibitor and is not metabolised by the Cytochrome P3A4 (CYP3A4) enzyme system, which is frequently inhibited by certain HAART therapies. The lack of requirement of CYP3A4 metabolism for rosuvastatin makes it an attractive lipid-lowering drug to treat the dyslipidemia associated with HAART [9]. To date only one study has examined the effect of rosuvastatin for treatment of HAART-related dyslipidemia wherein Calza et al determined that rosuvastatin effectively lowered total cholesterol (TC), LDL-cholesterol (LDL-C) and triglycerides (TG) while raising HDL-cholesterol (HDL-C) in HIV+ patients [10].

Elucidating any resistance to this new, potent statin would be of great clinical importance given the potential for wide use of rosuvastatin in this population, which is considered to be at moderate risk for cardiovascular disease.

## Results

### Lipid analysis

The overall effect of rosuvastatin on lipid parameters was as follows: TC decreased from 6.54 to 4.89 mmol/L (25.0% reduction, p < 0.001). Mean triglycerides decreased from 5.26 to 3.68 mmol/L (30.1% reduction, p < 0.001). Mean LDL decreased from 3.39 to 2.34 mmol/L (30.8% reduction, p < 0.001). Mean HDL rose from 1.04 to 1.06 mmol/L (2.0% increase, p = ns). Mean TC:HDL decreased from 7.22 to 5.03 (30.3%, p < 0.001). Mean apolipoprotein B decreased from 1.30 to 0.97 g/L (25.3% decrease, p < 0.001). The serum triglycerides of 33 patients were significantly elevated thus precluding calculation of serum LDL-C concentration. Five (5) patients experienced adverse events that ultimately led to discontinuation of the medication but were included in the study as they remained on the medication for longer than 4 weeks prior to being discontinued. Three (3) patients were discontinued due to elevated liver enzymes and 2 patients were discontinued due to complaints of muscle soreness.

### Dose-response analysis

**Figure 1:**
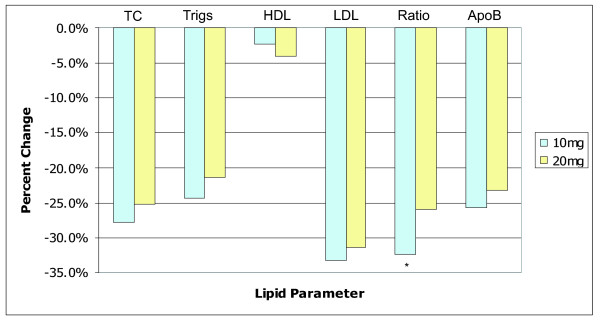
**Dose-response analysis**. Dose response analysis showing percentage change from baseline across all lipid parameters for 10 mg rosuvastatin monotherapy vs. 20 mg rosuvastatin monotherapy. * indicates p < 0.05.

A dose-response analysis was conducted wherein patients on rosuvastatin monotherapy (n = 68) were subdivided based on current rosuvastatin dosage. The 10 mg subgroup (n = 45) showed a greater improvement across all lipid parameters relative to the 20 mg subgroup (n = 23) with the exception of HDL (figure 1). Only the change in TC:HDL ratio was significant (p = 0.05). Patients taking rosuvastatin 5 mg (n = 1) or 40 mg (n = 1) were not included in this analysis.

### Combination therapy vs. monotherapy

**Figure 2:**
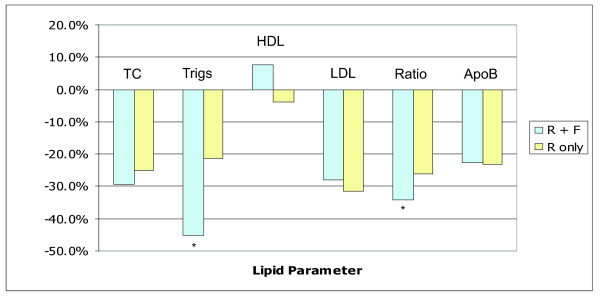
**Rosuvastatin monotherapy vs. Combination therapy**. The percentage changes from baseline in lipid parameters of HIV+ patients taking either Rosuvastatin (R) and Fenofibrate (F) combination therapy or Rosuvastatin monotherapy. * indicates p < 0.05.

Secondary analysis examined the effectiveness of a regime consisting of rosuvastatin as the sole lipid-lowering medication (rosuvastatin monotherapy, n = 70) vs. patients in whom rosuvastatin has been added onto their fenofibrate regime (combination therapy, n = 42) (Fig. 2). Patients on combination therapy showed a more significant drop in triglycerides (45.3% vs. 21.3%, p = 0.001) and TC:HDL (33.9% vs. 26.9%, p < 0.05) compared to patients on monotherapy. Additionally, patients on monotherapy showed a decrease in HDL of 4.1% compared to an increase of 7.6% in the combination therapy group (p = 0.08). All lipid parameters in both groups showed an improvement following addition of rosuvastatin when compared to baseline with the aforementioned exception of HDL in the monotherapy group.

## Discussion

While this study found that rosuvastatin is indeed effective at improving potentially atherogenic lipid parameters in HIV+ patients, an emerging theme from our results is an apparent resistance to therapy in our study population relative to previous studies involving non-HIV+ patients. Previous studies have shown statins and fibrates to be less effective in people with HIV compared to healthy subjects indicating possible resistance to lipid lowering therapy inherent to the virus itself or HAART [11,12]. As rosuvastatin has previously been shown to be effective in non-HIV populations both with and without the metabolic syndrome, when compared to other statins, we would expect an improvement of a similar magnitude in our study. However, this was not the case [13-20].

One large randomised clinical trial examining the effectiveness of rosuvastatin, in a non-HIV population, in comparison to other statins at improving plasma lipids noted improvements of -33.6% for TC, -46.7% for LDL-C, +9.3% for HDL-C and -23.4% for TG from baseline in patients with metabolic syndrome taking rosuvastatin (n = 240) [13]. By comparison in our study we saw changes from baseline of -25.1% (p < 0.001), -31.5% (p < 0.001), -4.1% (p < 0.05) and -21.3% for TC, LDL-C, HDL-C and TG respectively for patients on rosuvastatin monotherapy (n = 70). The most striking observations were the drop in HDL-C and the blunted improvement across all other lipid parameters.

The only other study examining the effects of rosuvastatin in an HIV+ population also showed improvements that were not as significant as would be expected from this highly potent statin. Calza et al randomised HIV+ patients to rosuvastatin or placebo for 24 weeks (n = 16) and reported median changes from baseline of -21.7% (TC), -22.4% (LDL-C) and -30.1% (TG) in atherogenic lipid lowering parameters, along with a 28.5% median increase in HDL [10]. These results are similar to ours with the exception of the drastic difference in change in HDL-C. This lack of effect of rosuvastatin on HDL is a novel finding in the face of previous studies who found that rosuvastatin improves HDL in both HIV and non-HIV populations [10,13]. In the present study the dyslipidemia is a drug-induced dyslipidemia the mechanism of which is still not well understood. The lack of effect on HDL may be related to an underlying cause of dyslipidemia induced by HIV itself.

Our dose response analysis (Fig. 1) showed very little difference between the 10 and 20 mg doses of rosuvastatin. Despite only the TC:HDL ratio showing a statistically significant improvement in the 10 mg group (-32.5% vs. -19.4%) these findings support the possibility of an underlying mechanism of resistance to statin therapy in HIV+ patients.

Resistance to statin therapy is not unique to rosuvastatin as evidenced by studies examining the effects of pravastatin in the HIV population. These studies observed no better than a 20.4% decrease in LDL-C compared to a 32.7% decrease in the general population. In each case the treatment group was given pravastatin 40 mg [21-23].

Protease inhibitor (PI)-containing HAART is often cited as a cause dyslipidemia and lipodystrophy in HIV patients and could possibly be the underlying cause of resistance to therapy observed in this study [1,2,4]. In the present study sub-analysis was conducted wherein patients were stratified according to their current HAART regimen and there did appear to be a less pronounced effect on lipid parameters, especially TC and LDL, in patients on PI-containing regimens. However there was no statistical significance between the groups for any given lipid measurement likely owing to the fact that the number of patients on non-PI-containing regimens (n = 14) was so much smaller than those on PI-containing regimens (n = 109) (data not shown). Other traditional explanations for the lack of effect rosuvastatin shown in our study include interaction with antacids, reduced compliance and lack of financial resources. These explanations, while valid in certain populations, are unlikely for the present study as all patients at our clinic are advised against taking antacids by the resident pharmacist. Furthermore the pharmacist reviews each patient's drug compliance every three months and has found that, generally, patients in our clinic are very compliant. Finally, in this center, the cost of rosuvastatin is covered by the provincial drug formulary and lack of financial resources should not play a role in patient compliance.

The current published guidelines recommend diet and exercise counselling, alteration of ARV regimen or adding lipid lowering medications for dyslipidemia in patients on ARV therapy [7]. A statin is suggested for LDL-C elevations and a fibrate is suggested for serum triglycerides elevation. In the face of possible resistance to rosuvastatin therapy in the HIV population a combination therapy approach of rosuvastatin plus fibrate may be a wise option to consider. The secondary analysis in this study assessed patients on combination therapy, specifically rosuvastatin with fenofibrate (Fig. 2). The combination therapy group showed an improvement in both triglycerides (45.3% vs. 23.4%, p < 0.05) and TC:HDL ratio (33.9% vs. 26.0%, p < 0.05) over the monotherapy group, with the latter driven by a non-significant difference in HDL. The addition of rosuvastatin to existing fenofibrate therapy seems promising especially given these improvements as well as the accompanying though non-significant increase in HDL-C in the combination therapy group. Although this increase HDL-C is modest (7.6%) it is more encouraging than the drop of 4.1% observed in the monotherapy group. While the improvements in the combination group can be perceived as less than expected it should be kept in mind that this study was observational in nature. Patients on combination therapy were likely treated as such because their dyslipidemia proved resistant to initial therapy and a more aggressive approach was deemed necessary.

## Conclusion

The resistance to therapy evidenced by the primary lipid analysis and the dose-response analysis of this study is of importance to clinicians treating HIV dyslipidemia as it demonstrates that simply increasing the dosage of rosuvastatin may not be the most effective way to improve lipid parameters in this population. Rosuvastatin is well suited for the treatment of HIV dyslipidemia as it is not metabolised by the CYP3A4 pathway, therefore, we suggest that clinicians consider a combination approach involving low dose rosuvastatin (i.e. 10 mg) in combination with either a fibrate or ezetimibe as data presented here and previously published data suggest that these combinations are effective [9,24].

## Methods

### Study design/Setting

A retrospective analysis was undertaken to determine the effect of rosuvastatin on the lipid profile of patients with HIV.

All patients were seen at the Immunodeficiency Clinic/HIV Metabolic Clinic (IDC/HIVMC) at St. Paul's Hospital, Vancouver, BC, Canada. This is a tertiary referral center where over 700 patients receive care for HIV associated metabolic disorders. Patients who were treated with rosuvastatin between January 2003 and July 2006 were included in the analysis. Rosuvastatin therapy was initiated either as an alternative to a previously prescribed statin that proved ineffective, either alone or in combination with other lipid lowering therapy, as an add-on to the patient's current lipid lowering therapy or as monotherapy to statin-naïve patients.

The effect of rosuvastatin on serum lipid concentrations of TC, LDL-C, HDL-C, TG and apolipoprotein B (Apo B) was analysed. Adverse events, as indicated by ALT or AST five times upper limit of normal (ULN) or CK ten times ULN as well as any condition requiring discontinuation of the drug, were documented.

### Patients

161 patients seen in the IDC/HIVMC had been prescribed rosuvastatin in dosages of 5,10,20 or 40 mg per day orally. 14 were excluded because exact start date of rosuvastatin could not be determined and an additional 17 were excluded because they lacked sufficient data for analysis and 130 patients remained for analysis. Five patients discontinued rosuvastatin therapy due to adverse events. For these patients their most recent lipid parameters prior to discontinuation were used in study analyses. Concomitant medications including other lipid lowering therapy, namely cholesterol transport blockers (ezetimibe) and fibrates (fenofibrate), and previous use of statins were documented as well as each patient's current antiretroviral regimen, simplified to either regimens containing protease inhibitors (PI) or those not containing protease inhibitors (Non-PI).

Mean patient age was 52.6 ± 8.29 years, 128 (98.5%) patients were male and 20 (15.4%) had diabetes mellitus. The mean duration of rosuvastatin therapy was 140.8 ± 112.84 days. 78 (60.0%) patients had previously taken a statin (64 atorvastatin, 10 pravastatin, 2 fluvastatin, 1 cerivastatin, 1 simvastatin) before being switched to rosuvastatin. 109 (83.8%) patients were on PI ARV regimens, 14 (10.8%) were on non-PI ARV regimens and 7 (5.4%) were taking no ARV therapy.

### Analysis/Ethics

Student's T-test for both paired and unpaired samples was used to compare pre and post treatment differences and subgroup differences, respectively.

Ethical approval was granted by the Research Ethics Board at St. Paul's Hospital.

## Competing interests

The author(s) declare that they have no competing interests.

## Authors' contributions

KJ was the principle author of the paper, participated in design of the project, performed statistical analysis and was the primary collector of data by means of chart review. MB participated in design of the project and edited the manuscript. GB designed the project, aided in data acquisition and was the principal editor of the manuscript. All authors read and approved the final manuscript.
